# Author Correction: Suicide among cancer patients

**DOI:** 10.1038/s41467-020-14506-7

**Published:** 2020-01-31

**Authors:** Nicholas G. Zaorsky, Ying Zhang, Leonard Tuanquin, Shirley M. Bluethmann, Henry S. Park, Vernon M. Chinchilli

**Affiliations:** 1 Department of Radiation Oncology, Penn State Cancer Institute, Hershey, PA 17033 USA; 2 Department of Public Health Sciences, Penn State College of Medicine, Hershey, PA 17033 USA; 3 Department of Therapeutic Radiology, Yale School of Medicine, Smilow Cancer Hospital at Yale, 35 Park Street, New Haven, CT 06511 USA

**Keywords:** Cancer, Cancer epidemiology, Human behaviour, Public health

Correction to: *Nature Communications* 10.1038/s41467-018-08170-1, published online 14 January 2019

In the original version of this Article the SEER database was described in Supplementary Note 1. This section has now been updated to include additional information, which was missing from the original file, and describes the registry differences, calculation of standardised mortality rates and latency exclusion periods in standardized mortality ratios and is entitled ‘Intricacies of Surveillance, Epidemiology, and End Results (SEER) Databases’.

