# Supplementary material for: Suicide among cancer patients
Source: Nat Commun. 2019 Jan 14;10:207. doi: 10.1038/s41467-018-08170-1 (PMC6331593; doi:10.1038/s41467-018-08170-1)
Supplement: Supplementary file 1 — SI file [file 41467_2018_8170_MOESM1_ESM.pdf]

SUPPLEMENTARY NOTE 1

Calendar period, registry, and diversity

The SEER program has evolved since its inception in the United States in 1973.[1] As of 2017, SEER has up to 36 years of longitudinal and ongoing data collection, with a representative sample size of more than 6 million cancer cases, and a comprehensive quality assurance process. Over time, more registries were added to SEER; in the current analysis, the SEER 18 (adjusted for Hurricane Katrina Impacted Louisiana cases) and SEER 9 registries were used. The registry number denotes the number of registries. *SEER 9.* The first areas included at that time were Connecticut, Hawaii, Iowa, San Francisco/Oakland, and Detroit. Geographic areas were included based on two objectives: (1) the ability of a geographic cancer registry to maintain high-quality data (explained below), and (2) having a population that represents minority subpopulations.[1]

In 1974-1975, the metropolitan areas of Atlanta and Seattle/Puget Sound were added, and the "SEER 9" registry was finalized. *SEER 11*. In order to expand on the second objective, two ore registries were added, Los Angeles County and 4 Counties in the San Jose/Monterey area. These counties included cases diagnosed after 1992. *SEER 13*. The next grouping additionally included 10 predominantly African American counties of rural Georgina and the Alaska Native American Tumor Registry. *SEER 17*. For cancers diagnoses after 2001, four additional areas were included: the remaining counties of California, Kentucky, Louisiana, and New Jersey. These counties have supplemental funding by the Centers for Disease Control (CDC). Based on the inclusion of these areas, the SEER database is representative of the population of the USA, and this has been validated by external studies.[1]

Since the SEER database have increased the proportion of the US population captured of the years, in early years of the SEER program there are fewer survivors than in later years, and the proportion of death by index cancer is lower in later years. Further, the "rate count" of people having a cancer depends on the number of patients living with this cancer from previous years (which depends on cancer prevalence), those diagnosed within the calendar year (which depends on screening and incidence), and those dying during that year (which depends on cancer and treatment aggressiveness, how death is coded, common risk factors among cancers and comorbidities, and patient age). Certain cancers have an indolent course (e.g. prostate), and patients diagnosed in subsequent years are added to the cumulative count, increasing the number of prostate cancer patients relative to all others; for patients with aggressive cancers (e.g. pancreatic), the addition of patients diagnosed in subsequent years has little effect on the cumulative number because of high rates of mortality.

Age

SEER provides age-standard adult (age ≥15) cancer populations to calculate age-standardized survival, which is used to compare survival across time or different cancer populations with different age distributions. The standards provided are the International Cancer Survival Standard (ICSS) derived for three broad groups of cancer sites with similar patterns of incidence by age. ICSS 1 includes cancer sites with increasing incidence by age (most cancer sites; e.g. prostate). ICSS 2 includes cancer sites with broadly constant incidence by age (e.g. nasopharynx). ICSS 3 includes cancer sites that mainly affect young adults (e.g. testis). By using the appropriate standard, the age-standardized survival is theorized to be like the raw (un-weighted) survival. For each of the three ICSS populations, SEER*Stat provides weights by 5-year age bins using the age variable, "Age recode with <1 year olds," and by five larger age

groups, in the variable, "Age Standard for Survival (15-44, 45-54, 55-64, 65-74, 75+)," as described on the SEER website.

Quality assurance and completeness

SEER undergoes quality assurance using systematic, standardized, and periodic data collection procedure for all defined members of a defined cohort is performed to avoid surveillance bias.[1] The case-finding audits are performed by a qualified member from each SEER registry under the direction of members of the National Cancer Institute. Auditors create an abstract the contains the primary site and the case finding source.[2] When performing audits, SEER adheres to two basic principles: auditing high quantity and high risk data. High quantity refers to disease sites that have the highest incidence and prevalence (e.g. breast, prostate, lung, colon); as well facilities that contribute the greatest percent of cases to the central database. Additionally, pathology laboratories are selected to review tissue from patients not seen at that hospital. High risk refers to cases that are likely to be miscoded (e.g. head and neck, hematopoietic diseases); compliance to new rules; and newly-reportable diseases.

Defining the cause of death

Mortality codes in SEER are assigned from death certificates, completed by the doctor caring for the patient at the time of demise. There is no single best method for calculating survival from cancer in the SEER program.[3] Different methods can give different outcomes, but for most variants considered the differences are small. For suicide, there is likely little discrepancy in the cause of death, as compared to a cause of death like heart disease, which may be cause by the cancer treatment, underlying heart disease, or a combination of both.

Intricacies of Surveillance, Epidemiology, and End Results (SEER) Databases

Registry Differences

The SEER databases have been evolving over the years, and this evolution is described in our Data Availability Statement and by previous work.[1,4-6] Briefly, SEER covers key demographic areas in the United States, and these areas/databases have slowly been added to SEER since the 1970s. The SEER 9 database includes 9 registries from 1973-2014; the SEER 18 database contains 18 registries, including the most recent patients from 2000-2016. Notably, SEER 18 is not limited to this time period; rather, the "2000-2016" refers to when all databases are collecting the data. Prior databases and their patients (before 2000) are available in SEER 18. The SEER 21 database was released in 2019, including more geographic regions. As data are collected from more regions, the same concepts of patient inclusion over time apply.

SEER is able to analyze data by different methods, using its "Sessions." The time period of these data sessions depend on the SEER database chosen (SEER 9, SEER 18, SEER 21, etc.). The "Standardized Incidence Ratio (SIR) session" provides incidence of a particular event after diagnosis, as a function of follow up time or age at diagnosis. When the event of interest is death as a function of follow up time, the SIRs are actually standardized mortality ratios (SMRs), and they provide the relative risk of death from a particular cause vs. the general population.

A "case listing" session is another option in displaying the data. Case listing sessions provide patient-level data, with each patient in a row, and variables (e.g. age, sex, cancer type) in columns. Thus, case listing sessions may be used to calculate odds ratios and generate survival plots.

Calculating Standardized Mortality Ratios

SMRs consist of two measures: (observed number of events, during time at risk) / (expected number of events in the reference population, during time at risk). SMRs may be calculated as a function of different times at risk, including time after diagnosis (i.e. the latency period) or age at diagnosis. When SMRs are calculated as a function of time after diagnosis, they provide the relative risk of death from one particular cause vs. the reference population. The reference population changes depending on the population and the time period. Thus, SMRs should not be compared to one another, and they would be expected to vary over different time periods or with different patient populations. Further, calculated SMRs may differ when using different SEER databases because (1) the observed number of events of interest among cancer patients may change, and (2) the number of events of interest in the reference population (i.e. the United States) also changes over the years.

Latency Exclusion Periods in Standardized Mortality Ratios

For SMRs calculated as a function of follow up time, SMRs during each window of time (e.g. at 1 year after diagnosis, 1-5 years after diagnosis, etc.) depend on the time at risk. With longer time at risk and more observed events, the confidence intervals become smaller, and measurements are more accurate. With a short time at risk (e.g. the first few months after diagnosis), or very few events (e.g. suicide), or among a niche patient cohort (e.g. Hodgkin lymphoma), the confidence intervals can widen dramatically.

In the first few months after diagnosis of cancer, patients often have an “introduction to the medical system;” i.e. a patient living in a rural area comes to a hospital where they are diagnosed with cancer, as well as many other comorbidities like heart disease, lung dysfunction, kidney failure, etc. The patient may die of any of these within a few months, but estimating the observed versus expected rate of death becomes difficult, and the confidence intervals for an

SMR naturally widen. Thus, some researchers, including our team, sometimes elect to exclude the first 2 months from the SMR calculations. While SMRs may actually be very high during this time, the confidence intervals are so wide that an accurate measure is not meaningful. Moreover, the absolute number of observed events in this time may be rather low, especially when the event of interest is rare. Thus, the overall SMRs for the entire follow up period (with or without the latency periods) tend to be relatively similar.

SUPPLEMENTARY REFERENCES

1 Park, H. S., Lloyd, S., Decker, R. H., Wilson, L. D. & Yu, J. B. Overview of the Surveillance, Epidemiology, and End Results database: evolution, data variables, and quality assurance. *Curr. Probl. Cancer* **36**, 183-190, doi:10.1016/j.currproblcancer.2012.03.007 (2012).

2 *National Cancer Institute. Casefinding Studies - SEER Quality Improvement.*, <http://seer.cancer.gov/qi/tools/casefinding.html> (2016).

3 Boer, R. *et al.* (Statistical Research and Applications Branch, NCI, Bethesda, MD).

4 Park, H. S., Lloyd, S., Decker, R. H., Wilson, L. D. & Yu, J. B. Limitations and biases of the Surveillance, Epidemiology, and End Results database. *Curr. Probl. Cancer* **36**, 216-224, doi:10.1016/j.currproblcancer.2012.03.011 (2012).

5 Zaorsky, N. G. *et al.* Suicide among cancer patients. *Nature communications* **10**, 207, doi:10.1038/s41467-018-08170-1 (2019).

6 Zaorsky, N. G. *et al.* Causes of death among cancer patients. *Ann. Oncol.* **28**, 400-407, doi:10.1093/annonc/mdw604 (2017).

SUPPLEMENTARY METHODS

Instructions to access the SEER data.

(1) Download the SEER*Stat software from the NCI website: https://seer.cancer.gov/seerstat/software/

(2) Open the program

(3) Click “File”, “New,”

“MP-SIR” Session to generate the SMRs. Note, this was used in Table 1 and Figure 1 of the current analysis.

“Case Listing” to generate a list of patient cases diagnosed.

“Incidence” to generate a list of the incidence of cancer or cause of death. Note, this was used in Table 2 and Figure 2 of the current analysis, and to generate the ORs.

(4) Click on the desired registry to use for each of the sessions. For the purposes of this analysis, the following registries and options were selected. All the other data supporting the findings of this study are available within the article and its supplementary information files and from the corresponding author upon reasonable request

In the “MP-SIR” session, select the following:

Filename: SIR suicide Matrix-2

SEER*Sta
t Version: 8.3.4
Date: ########

Session Type: MP-SIR
SUGGESTED CITATION
Software: Surveillance Research Program, National Cancer Institute SEER*Stat software (www.seer.cancer.gov/seerstat) version 8.3.4.
Data: Surveillance, Epidemiology, and End Results (SEER) Program (www.seer.cancer.gov) SEER*Stat Database: Incidence - SEER 9 Regs Research Data,
Nov 2016 Sub (1973-2014) for SMRs - Linked To County Attributes - Total U.S., 1969-2015 Counties, National Cancer Institute, DCCPS, Surveillance
Research Program, released April 2017, based on the November 2016 submission.
DATA
Database: Incidence - SEER 9 Regs Research Data, Nov 2016 Sub (1973-2014) for SMRs - Linked To County Attributes - Total U.S., 1969-2015 Counties
RATES
Name: U.S. Mortality 1973-2014 (Nov 2016 sub), Race (WU/B/O), Event: COD rec (HIV grouped w/oth infectious)
See rate session page(s) below.
SELECTION
Index Record: First Primary Only (Sequence Number 0 or 1)
Select Only: Malignant Cases, Known Age
Exclude All Death Certificate and Autopsy Only
PARAMETERS
Exposure Date: Date of diagnosis recode
Latency Exclusion: 2 months
Start Date: Date of diagnosis recode
Cutoff Start: Jan 1973
Cutoff End: Dec 2014
Latency: 1y,5y
EVENTS
Analysis Type: Single Outcome Analysis
Exit Point: Exit at Any Event in Rate File
Event possible on Index Record
Early Exit: Next Malignant Tumor
Selected Events: COD rec (HIV grouped w/oth infectious) (Event Variable)
All Causes of Death
All Malignant Cancers
Oral Cavity and Pharynx
Lip
Tongue
Salivary Gland
Floor of Mouth
Gum and Other Mouth
Nasopharynx
Tonsil
Oropharynx
Hypopharynx
Other Oral Cavity and Pharynx
Digestive System
Esophagus
Stomach
Small Intestine
Colon and Rectum

Colon excluding Rectum
Rectum and Rectosigmoid Junction
Anus, Anal Canal and Anorectum
Liver and Intrahepatic Bile Duct
Liver
Intrahepatic Bile Duct
Gallbladder
Other Biliary
Pancreas
Retroperitoneum
Peritoneum, Omentum and Mesentery
Other Digestive Organs
Respiratory System
Nose, Nasal Cavity and Middle Ear
Larynx
Lung and Bronchus
Pleura
Trachea, Mediastinum and Other Respiratory Organs
Bones and Joints
Soft Tissue including Heart
Skin excluding Basal and Squamous
Melanoma of the Skin
Other Non-Epithelial Skin
Breast
Female Genital System
Cervix Uteri
Corpus and Uterus, NOS
Corpus Uteri
Uterus, NOS
Ovary
Vagina
Vulva
Other Female Genital Organs
Male Genital System
Prostate
Testis
Penis
Other Male Genital Organs
Urinary System
Urinary Bladder
Kidney and Renal Pelvis
Ureter
Other Urinary Organs
Eye and Orbit
Brain and Other Nervous System
Endocrine System
Thyroid
Other Endocrine including Thymus
Lymphoma
Hodgkin Lymphoma
Non-Hodgkin Lymphoma
Myeloma
Leukemia
Lymphocytic Leukemia
Acute Lymphocytic Leukemia
Chronic Lymphocytic Leukemia

Other Lymphocytic Leukemia
Myeloid and Monocytic Leukemia
Acute Myeloid Leukemia
Acute Monocytic Leukemia
Chronic Myeloid Leukemia
Other Myeloid/Monocytic Leukemia
Other Leukemia
Other Acute Leukemia
Aleukemic, Subleukemic and NOS
Miscellaneous Malignant Cancer
In situ, benign or unknown behavior neoplasm
Tuberculosis
Syphilis
Septicemia
Other Infectious and Parasitic Diseases including HIV
a - Do not use, not compatible with SEER incidence
b - Do not use, not compatible with SEER incidence
Diabetes Mellitus
Alzheimers (ICD-9 and 10 only)
Diseases of Heart
Hypertension without Heart Disease
Cerebrovascular Diseases
Atherosclerosis
Aortic Aneurysm and Dissection
Other Diseases of Arteries, Arterioles, Capillaries
Pneumonia and Influenza
Chronic Obstructive Pulmonary Disease and Allied Cond
Stomach and Duodenal Ulcers
Chronic Liver Disease and Cirrhosis
Nephritis, Nephrotic Syndrome and Nephrosis
Complications of Pregnancy, Childbirth, Puerperium
Congenital Anomalies
Certain Conditions Originating in Perinatal Period
Symptoms, Signs and Ill-Defined Conditions
Accidents and Adverse Effects
Suicide and Self-Inflicted Injury
Homicide and Legal Intervention
Other Cause of Death

STATISTICS
Table Type: SIR Tables
Include: 95% Confidence Intervals (Exact Method)
TABLE
Page: COD to site recode
Row: Site recode B ICD-O-3/WHO 2008
Column: Latency [*calculated*]
USER DEFINITIONS

COD rec (HIV grouped w/oth infectious) (Event Variable) [Cause of death recode]
All Causes of Death = Lip, Tongue, Salivary Gland, Floor of Mouth, Gum and Other Mouth, Nasopharynx, Tonsil, Oropharynx, Hypopharynx, Other Oral Cavity and Pharynx, Esophagus, Stomach, Small Intestine, Colon excluding Rectum, Rectum and Rectosigmoid Junction, Anus, Anal Canal and Anorectum, Liver, Intrahepatic Bile Duct, Gallbladder, Other Biliary, Pancreas, Retroperitoneum, Peritoneum, Omentum and Mesentery, Other Digestive Organs, Nose, Nasal Cavity and Middle Ear, Larynx, Lung and Bronchus, Pleura, Trachea, Mediastinum and Other Respiratory Organs, Bones and Joints,

Soft Tissue including Heart, Melanoma of the Skin, Non-Melanoma Skin, Breast, Cervix Uteri, Corpus Uteri, Uterus, NOS, Ovary, Vagina, Vulva, Other
Female Genital Organs, Prostate, Testis, Penis, Other Male Genital Organs, Urinary Bladder, Kidney and Renal Pelvis, Ureter, Other Urinary Organs,
Eye and Orbit, Brain and Other Nervous System, Thyroid, Other Endocrine including Thymus, Hodgkin Lymphoma, Non-Hodgkin Lymphoma, Myeloma,
Acute Lymphocytic Leukemia, Chronic Lymphocytic Leukemia, Other Lymphocytic Leukemia, Acute Myeloid Leukemia, Acute Monocytic Leukemia,
Chronic Myeloid Leukemia, Other Myeloid/Monocytic Leukemia, Other Acute Leukemia, Aleukemic, Subleukemic and NOS, Miscellaneous Malignant
Cancer, In situ, benign or unknown behavior neoplasm, Tuberculosis, Syphilis, 139, Septicemia, Other Infectious and Parasitic Diseases including HIV,
Diabetes Mellitus, Alzheimers (ICD-9 and 10 only), Diseases of Heart, Hypertension without Heart Disease, Cerebrovascular Diseases, Atherosclerosis,
Aortic Aneurysm and Dissection, Other Diseases of Arteries, Arterioles, Capillaries, Pneumonia and Influenza, Chronic Obstructive Pulmonary Disease and
Allied Cond, Stomach and Duodenal Ulcers, Chronic Liver Disease and Cirrhosis, Nephritis, Nephrotic Syndrome and Nephrosis, Complications of
Pregnancy, Childbirth, Puerperium, Congenital Anomalies, Certain Conditions Originating in Perinatal Period, Symptoms, Signs and Ill-Defined Conditions,
Accidents and Adverse Effects, Suicide and Self-Inflicted Injury, Homicide and Legal Intervention, Other Cause of Death

All Malignant Cancers = Lip, Tongue, Salivary Gland, Floor of Mouth, Gum and Other Mouth, Nasopharynx, Tonsil, Oropharynx, Hypopharynx, Other Oral
Cavity and Pharynx, Esophagus, Stomach, Small Intestine, Colon excluding Rectum, Rectum and Rectosigmoid Junction, Anus, Anal Canal and
Anorectum, Liver, Intrahepatic Bile Duct, Gallbladder, Other Biliary, Pancreas, Retroperitoneum, Peritoneum, Omentum and Mesentery, Other Digestive
Organs, Nose, Nasal Cavity and Middle Ear, Larynx, Lung and Bronchus, Pleura, Trachea, Mediastinum and Other Respiratory Organs, Bones and Joints,
Soft Tissue including Heart, Melanoma of the Skin, Non-Melanoma Skin, Breast, Cervix Uteri, Corpus Uteri, Uterus, NOS, Ovary, Vagina, Vulva, Other
Female Genital Organs, Prostate, Testis, Penis, Other Male Genital Organs, Urinary Bladder, Kidney and Renal Pelvis, Ureter, Other Urinary Organs,
Eye and Orbit, Brain and Other Nervous System, Thyroid, Other Endocrine including Thymus, Hodgkin Lymphoma, Non-Hodgkin Lymphoma, Myeloma,
Acute Lymphocytic Leukemia, Chronic Lymphocytic Leukemia, Other Lymphocytic Leukemia, Acute Myeloid Leukemia, Acute Monocytic Leukemia,
Chronic Myeloid Leukemia, Other Myeloid/Monocytic Leukemia, Other Acute Leukemia, Aleukemic, Subleukemic and NOS, Miscellaneous Malignant Cancer

Oral Cavity and Pharynx = Lip, Tongue, Salivary Gland, Floor of Mouth, Gum and Other Mouth, Nasopharynx, Tonsil, Oropharynx, Hypopharynx, Other
Oral Cavity and Pharynx

Lip = Lip

Tongue = Tongue

Salivary Gland = Salivary Gland

Floor of Mouth = Floor of Mouth

Gum and Other Mouth = Gum and Other Mouth

Nasopharynx = Nasopharynx

Tonsil = Tonsil

Oropharynx = Oropharynx

Hypopharynx = Hypopharynx

Other Oral Cavity and Pharynx = Other Oral Cavity and Pharynx

Digestive System = Esophagus, Stomach, Small Intestine, Colon excluding Rectum, Rectum and Rectosigmoid Junction, Anus, Anal Canal and Anorectum,
Liver, Intrahepatic Bile Duct, Gallbladder, Other Biliary, Pancreas, Retroperitoneum,

Peritoneum, Omentum and Mesentery, Other Digestive Organs
  Esophagus = Esophagus
  Stomach = Stomach
  Small Intestine = Small Intestine
  Colon and Rectum = Colon excluding Rectum, Rectum and Rectosigmoid Junction
  Colon excluding Rectum = Colon excluding Rectum
  Rectum and Rectosigmoid Junction = Rectum and Rectosigmoid Junction
  Anus, Anal Canal and Anorectum = Anus, Anal Canal and Anorectum
  Liver and Intrahepatic Bile Duct = Liver, Intrahepatic Bile Duct
  Liver = Liver
  Intrahepatic Bile Duct = Intrahepatic Bile Duct
  Gallbladder = Gallbladder
  Other Biliary = Other Biliary
  Pancreas = Pancreas
  Retroperitoneum = Retroperitoneum
  Peritoneum, Omentum and Mesentery = Peritoneum, Omentum and Mesentery
  Other Digestive Organs = Other Digestive Organs
  Respiratory System = Nose, Nasal Cavity and Middle Ear, Larynx, Lung and Bronchus, Pleura, Trachea, Mediastinum and Other Respiratory Organs
  Nose, Nasal Cavity and Middle Ear = Nose, Nasal Cavity and Middle Ear
  Larynx = Larynx
  Lung and Bronchus = Lung and Bronchus
  Pleura = Pleura
  Trachea, Mediastinum and Other Respiratory Organs = Trachea, Mediastinum and Other Respiratory Organs
  Bones and Joints = Bones and Joints
  Soft Tissue including Heart = Soft Tissue including Heart
  Skin excluding Basal and Squamous = Melanoma of the Skin, Non-Melanoma Skin
  Melanoma of the Skin = Melanoma of the Skin
  Other Non-Epithelial Skin = Non-Melanoma Skin
  Breast = Breast
  Female Genital System = Cervix Uteri, Corpus Uteri, Uterus, NOS, Ovary, Vagina, Vulva, Other Female Genital Organs
  Cervix Uteri = Cervix Uteri
  Corpus and Uterus, NOS = Corpus Uteri, Uterus, NOS
  Corpus Uteri = Corpus Uteri
  Uterus, NOS = Uterus, NOS
  Ovary = Ovary
  Vagina = Vagina
  Vulva = Vulva
  Other Female Genital Organs = Other Female Genital Organs
  Male Genital System = Prostate, Testis, Penis, Other Male Genital Organs
  Prostate = Prostate
  Testis = Testis
  Penis = Penis
  Other Male Genital Organs = Other Male Genital Organs
  Urinary System = Urinary Bladder, Kidney and Renal Pelvis, Ureter, Other Urinary Organs
  Urinary Bladder = Urinary Bladder
  Kidney and Renal Pelvis = Kidney and Renal Pelvis
  Ureter = Ureter
  Other Urinary Organs = Other Urinary Organs
  Eye and Orbit = Eye and Orbit
  Brain and Other Nervous System = Brain and Other Nervous System
  Endocrine System = Thyroid, Other Endocrine including Thymus
  Thyroid = Thyroid

Other Endocrine including Thymus = Other Endocrine including Thymus
Lymphoma = Hodgkin Lymphoma, Non-Hodgkin Lymphoma
Hodgkin Lymphoma = Hodgkin Lymphoma
Non-Hodgkin Lymphoma = Non-Hodgkin Lymphoma
Myeloma = Myeloma
Leukemia = Acute Lymphocytic Leukemia, Chronic Lymphocytic Leukemia, Other Lymphocytic Leukemia, Acute Myeloid Leukemia, Acute Monocytic Leukemia, Chronic Myeloid Leukemia, Other Myeloid/Monocytic Leukemia, Other Acute Leukemia, Aleukemic, Subleukemic and NOS
Lymphocytic Leukemia = Acute Lymphocytic Leukemia, Chronic Lymphocytic Leukemia, Other Lymphocytic Leukemia
Acute Lymphocytic Leukemia = Acute Lymphocytic Leukemia
Chronic Lymphocytic Leukemia = Chronic Lymphocytic Leukemia
Other Lymphocytic Leukemia = Other Lymphocytic Leukemia
Myeloid and Monocytic Leukemia = Acute Myeloid Leukemia, Acute Monocytic Leukemia, Chronic Myeloid Leukemia, Other Myeloid/Monocytic Leukemia
Acute Myeloid Leukemia = Acute Myeloid Leukemia
Acute Monocytic Leukemia = Acute Monocytic Leukemia
Chronic Myeloid Leukemia = Chronic Myeloid Leukemia
Other Myeloid/Monocytic Leukemia = Other Myeloid/Monocytic Leukemia
Other Leukemia = Other Acute Leukemia, Aleukemic, Subleukemic and NOS
Other Acute Leukemia = Other Acute Leukemia
Aleukemic, Subleukemic and NOS = Aleukemic, Subleukemic and NOS
Miscellaneous Malignant Cancer = Miscellaneous Malignant Cancer
In situ, benign or unknown behavior neoplasm = In situ, benign or unknown behavior neoplasm
Tuberculosis = Tuberculosis
Syphilis = Syphilis
Septicemia = Septicemia
Other Infectious and Parasitic Diseases including HIV = 139, Other Infectious and Parasitic Diseases including HIV
a - Do not use, not compatible with SEER incidence = 139
b - Do not use, not compatible with SEER incidence = Other Infectious and Parasitic Diseases including HIV
Diabetes Mellitus = Diabetes Mellitus
Alzheimers (ICD-9 and 10 only) = Alzheimers (ICD-9 and 10 only)
Diseases of Heart = Diseases of Heart
Hypertension without Heart Disease = Hypertension without Heart Disease
Cerebrovascular Diseases = Cerebrovascular Diseases
Atherosclerosis = Atherosclerosis
Aortic Aneurysm and Dissection = Aortic Aneurysm and Dissection
Other Diseases of Arteries, Arterioles, Capillaries = Other Diseases of Arteries, Arterioles, Capillaries
Pneumonia and Influenza = Pneumonia and Influenza
Chronic Obstructive Pulmonary Disease and Allied Cond = Chronic Obstructive Pulmonary Disease and Allied Cond
Stomach and Duodenal Ulcers = Stomach and Duodenal Ulcers
Chronic Liver Disease and Cirrhosis = Chronic Liver Disease and Cirrhosis
Nephritis, Nephrotic Syndrome and Nephrosis = Nephritis, Nephrotic Syndrome and Nephrosis
Complications of Pregnancy, Childbirth, Puerperium = Complications of Pregnancy, Childbirth, Puerperium
Congenital Anomalies = Congenital Anomalies
Certain Conditions Originating in Perinatal Period = Certain Conditions Originating in Perinatal Period
Symptoms, Signs and Ill-Defined Conditions = Symptoms, Signs and Ill-Defined Conditions

Accidents and Adverse Effects = Accidents and Adverse Effects
Suicide and Self-Inflicted Injury = Suicide and Self-Inflicted Injury
Homicide and Legal Intervention = Homicide and Legal Intervention
Other Cause of Death = Other Cause of Death
State DC not available or state DC available but no COD = State DC not available or state DC available but no COD
All Events (system added) = Lip, Tongue, Salivary Gland, Floor of Mouth, Gum and Other Mouth, Nasopharynx, Tonsil, Oropharynx, Hypopharynx, Other Oral Cavity and Pharynx, Esophagus, Stomach, Small Intestine, Colon excluding Rectum, Rectum and Rectosigmoid Junction, Anus, Anal Canal and Anorectum, Liver, Intrahepatic Bile Duct, Gallbladder, Other Biliary, Pancreas, Retroperitoneum, Peritoneum, Omentum and Mesentery, Other Digestive Organs, Nose, Nasal Cavity and Middle Ear, Larynx, Lung and Bronchus, Pleura, Trachea, Mediastinum and Other Respiratory Organs, Bones and Joints, Soft Tissue including Heart, Melanoma of the Skin, Non-Melanoma Skin, Breast, Cervix Uteri, Corpus Uteri, Uterus, NOS, Ovary, Vagina, Vulva, Other Female Genital Organs, Prostate, Testis, Penis, Other Male Genital Organs, Urinary Bladder, Kidney and Renal Pelvis, Ureter, Other Urinary Organs, Eye and Orbit, Thyroid, Other Endocrine including Thymus, Hodgkin Lymphoma, Non-Hodgkin Lymphoma, Myeloma, Acute Lymphocytic Leukemia, Chronic Lymphocytic Leukemia, Other Lymphocytic Leukemia, Acute Myeloid Leukemia, Chronic Myeloid Leukemia, Acute Monocytic Leukemia, Other Acute Leukemia, Aleukemic, Subleukemic and NOS, Miscellaneous Malignant Cancer, Other Myeloid/Monocytic Leukemia, Brain and Other Nervous System, In situ, benign or unknown behavior neoplasm, Tuberculosis, Syphilis, 139, Septicemia, Other Infectious and Parasitic Diseases including HIV, Diabetes Mellitus, Alzheimers (ICD-9 and 10 only), Diseases of Heart, Hypertension without Heart Disease, Cerebrovascular Diseases, Atherosclerosis, Aortic Aneurysm and Dissection, Other Diseases of Arteries, Arterioles, Capillaries, Pneumonia and Influenza, Chronic Obstructive Pulmonary Disease and Allied Cond, Stomach and Duodenal Ulcers, Chronic Liver Disease and Cirrhosis, Nephritis, Nephrotic Syndrome and Nephrosis, Complications of Pregnancy, Childbirth, Puerperium, Congenital Anomalies, Certain Conditions Originating in Perinatal Period, Symptoms, Signs and Ill-Defined Conditions, Accidents and Adverse Effects, Suicide and Self-Inflicted Injury, Homicide and Legal Intervention, Other Cause of Death, State DC not available or state DC available but no COD

OUTPUT
Precision: 2 decimal places (default)
Historic: Not Selected
Rates: Rate Session

CITATION
Data: Surveillance, Epidemiology, and End Results (SEER) Program (www.seer.cancer.gov) SEER*Stat Database: Mortality - All COD, Aggregated Total U.S. (1969-2014) <Katrina/Rita Population Adjustment>, National Cancer Institute, DCCPS, Surveillance Research Program, Surveillance Systems Branch, released December 2016. Underlying mortality data provided by NCHS (www.cdc.gov/nchs).

DATA
Database: Mortality - All COD, Aggregated Total U.S. (1969-2014) <Katrina/Rita Population Adjustment>

STATISTIC
Statistic: Crude Rates

SELECTION
Select Only: Known Age

TABLE

Page: COD rec (HIV grouped w/oth infectious) [Cause of death recode]
Sex M/F [Sex]
Race (W/Unspec, B, O) [Race recode (White, Black, Other)]
Row: Age recode MPS [Age recode with <1 year olds]
Column: Year of death (1973-2014) [Year of death]
USER DEFINITIONS
COD rec (HIV grouped w/oth infectious) [Cause of death recode]
Description: For more information, see http://seer.cancer.gov/codrecode/1969+_d04162012.
All Causes of Death = Lip, Tongue, Salivary Gland, Floor of Mouth, Gum and Other Mouth, Nasopharynx, Tonsil, Oropharynx, Hypopharynx, Other Oral Cavity and Pharynx, Esophagus, Stomach, Small Intestine, Colon excluding Rectum, Rectum and Rectosigmoid Junction, Anus, Anal Canal and Anorectum, Liver, Intrahepatic Bile Duct, Gallbladder, Other Biliary, Pancreas, Retroperitoneum, Peritoneum, Omentum and Mesentery, Other Digestive Organs, Nose, Nasal Cavity and Middle Ear, Larynx, Lung and Bronchus, Pleura, Trachea, Mediastinum and Other Respiratory Organs, Bones and Joints, Soft Tissue including Heart, Melanoma of the Skin, Non-Melanoma Skin, Breast, Cervix Uteri, Corpus Uteri, Uterus, NOS, Ovary, Vagina, Vulva, Other Female Genital Organs, Prostate, Testis, Penis, Other Male Genital Organs, Urinary Bladder, Kidney and Renal Pelvis, Ureter, Other Urinary Organs, Eye and Orbit, Brain and Other Nervous System, Thyroid, Other Endocrine including Thymus, Hodgkin Lymphoma, Non-Hodgkin Lymphoma, Myeloma, Acute Lymphocytic Leukemia, Chronic Lymphocytic Leukemia, Other Lymphocytic Leukemia, Acute Myeloid Leukemia, Acute Monocytic Leukemia, Chronic Myeloid Leukemia, Other Myeloid/Monocytic Leukemia, Other Acute Leukemia, Aleukemic, Subleukemic and NOS, Miscellaneous Malignant Cancer, In situ, benign or unknown behavior neoplasm, Tuberculosis, Syphilis, Human Immunodeficiency Virus (HIV) (1987+), Septicemia, Other Infectious and Parasitic Diseases, Diabetes Mellitus, Alzheimers (ICD-9 and 10 only), Diseases of Heart, Hypertension without Heart Disease, Cerebrovascular Diseases, Atherosclerosis, Aortic Aneurysm and Dissection, Other Diseases of Arteries, Arterioles, Capillaries, Pneumonia and Influenza, Chronic Obstructive Pulmonary Disease and Allied Cond, Stomach and Duodenal Ulcers, Chronic Liver Disease and Cirrhosis, Nephritis, Nephrotic Syndrome and Nephrosis, Complications of Pregnancy, Childbirth, Puerperium, Congenital Anomalies, Certain Conditions Originating in Perinatal Period, Symptoms, Signs and Ill-Defined Conditions, Accidents and Adverse Effects, Suicide and Self-Inflicted Injury, Homicide and Legal Intervention, Other Cause of Death
All Malignant Cancers = Lip, Tongue, Salivary Gland, Floor of Mouth, Gum and Other Mouth, Nasopharynx, Tonsil, Oropharynx, Hypopharynx, Other Oral Cavity and Pharynx, Esophagus, Stomach, Small Intestine, Colon excluding Rectum, Rectum and Rectosigmoid Junction, Anus, Anal Canal and Anorectum, Liver, Intrahepatic Bile Duct, Gallbladder, Other Biliary, Pancreas, Retroperitoneum, Peritoneum, Omentum and Mesentery, Other Digestive Organs, Nose, Nasal Cavity and Middle Ear, Larynx, Lung and Bronchus, Pleura, Trachea, Mediastinum and Other Respiratory Organs, Bones and Joints, Soft Tissue including Heart, Melanoma of the Skin, Non-Melanoma Skin, Breast, Cervix Uteri, Corpus Uteri, Uterus, NOS, Ovary, Vagina, Vulva, Other Female Genital Organs, Prostate, Testis, Penis, Other Male Genital Organs, Urinary Bladder, Kidney and Renal Pelvis, Ureter, Other Urinary Organs, Eye and Orbit, Brain and Other Nervous System, Thyroid, Other Endocrine including Thymus, Hodgkin Lymphoma, Non-Hodgkin Lymphoma, Myeloma, Acute Lymphocytic Leukemia, Chronic Lymphocytic Leukemia, Other Lymphocytic Leukemia, Acute Myeloid Leukemia, Acute Monocytic Leukemia,

Chronic Myeloid Leukemia, Other Myeloid/Monocytic Leukemia, Other Acute Leukemia, Aleukemic, Subleukemic and NOS, Miscellaneous Malignant Cancer
Oral Cavity and Pharynx = Lip, Tongue, Salivary Gland, Floor of Mouth, Gum and Other Mouth, Nasopharynx, Tonsil, Oropharynx, Hypopharynx, Other Oral Cavity and Pharynx
Lip = Lip
Tongue = Tongue
Salivary Gland = Salivary Gland
Floor of Mouth = Floor of Mouth
Gum and Other Mouth = Gum and Other Mouth
Nasopharynx = Nasopharynx
Tonsil = Tonsil
Oropharynx = Oropharynx
Hypopharynx = Hypopharynx
Other Oral Cavity and Pharynx = Other Oral Cavity and Pharynx
Digestive System = Esophagus, Stomach, Small Intestine, Colon excluding Rectum, Rectum and Rectosigmoid Junction, Anus, Anal Canal and Anorectum, Liver, Intrahepatic Bile Duct, Gallbladder, Other Biliary, Pancreas, Retroperitoneum, Peritoneum, Omentum and Mesentery, Other Digestive Organs
Esophagus = Esophagus
Stomach = Stomach
Small Intestine = Small Intestine
Colon and Rectum = Colon excluding Rectum, Rectum and Rectosigmoid Junction
Colon excluding Rectum = Colon excluding Rectum
Rectum and Rectosigmoid Junction = Rectum and Rectosigmoid Junction
Anus, Anal Canal and Anorectum = Anus, Anal Canal and Anorectum
Liver and Intrahepatic Bile Duct = Liver, Intrahepatic Bile Duct
Liver = Liver
Intrahepatic Bile Duct = Intrahepatic Bile Duct
Gallbladder = Gallbladder
Other Biliary = Other Biliary
Pancreas = Pancreas
Retroperitoneum = Retroperitoneum
Peritoneum, Omentum and Mesentery = Peritoneum, Omentum and Mesentery
Other Digestive Organs = Other Digestive Organs
Respiratory System = Nose, Nasal Cavity and Middle Ear, Larynx, Lung and Bronchus, Pleura, Trachea, Mediastinum and Other Respiratory Organs
Nose, Nasal Cavity and Middle Ear = Nose, Nasal Cavity and Middle Ear
Larynx = Larynx
Lung and Bronchus = Lung and Bronchus
Pleura = Pleura
Trachea, Mediastinum and Other Respiratory Organs = Trachea, Mediastinum and Other Respiratory Organs
Bones and Joints = Bones and Joints
Soft Tissue including Heart = Soft Tissue including Heart
Skin excluding Basal and Squamous = Melanoma of the Skin, Non-Melanoma Skin
Melanoma of the Skin = Melanoma of the Skin
Other Non-Epithelial Skin = Non-Melanoma Skin
Breast = Breast
Female Genital System = Cervix Uteri, Corpus Uteri, Uterus, NOS, Ovary, Vagina, Vulva, Other Female Genital Organs
Cervix Uteri = Cervix Uteri
Corpus and Uterus, NOS = Corpus Uteri, Uterus, NOS
Corpus Uteri = Corpus Uteri
Uterus, NOS = Uterus, NOS
Ovary = Ovary

Vagina = Vagina
Vulva = Vulva
Other Female Genital Organs = Other Female Genital Organs
Male Genital System = Prostate, Testis, Penis, Other Male Genital Organs
Prostate = Prostate
Testis = Testis
Penis = Penis
Other Male Genital Organs = Other Male Genital Organs
Urinary System = Urinary Bladder, Kidney and Renal Pelvis, Ureter, Other Urinary Organs
Urinary Bladder = Urinary Bladder
Kidney and Renal Pelvis = Kidney and Renal Pelvis
Ureter = Ureter
Other Urinary Organs = Other Urinary Organs
Eye and Orbit = Eye and Orbit
Brain and Other Nervous System = Brain and Other Nervous System
Endocrine System = Thyroid, Other Endocrine including Thymus
Thyroid = Thyroid
Other Endocrine including Thymus = Other Endocrine including Thymus
Lymphoma = Hodgkin Lymphoma, Non-Hodgkin Lymphoma
Hodgkin Lymphoma = Hodgkin Lymphoma
Non-Hodgkin Lymphoma = Non-Hodgkin Lymphoma
Myeloma = Myeloma
Leukemia = Acute Lymphocytic Leukemia, Chronic Lymphocytic Leukemia, Other Lymphocytic Leukemia, Acute Myeloid Leukemia, Acute Monocytic Leukemia, Chronic Myeloid Leukemia, Other Myeloid/Monocytic Leukemia, Other Acute Leukemia, Aleukemic, Subleukemic and NOS
Lymphocytic Leukemia = Acute Lymphocytic Leukemia, Chronic Lymphocytic Leukemia, Other Lymphocytic Leukemia
Acute Lymphocytic Leukemia = Acute Lymphocytic Leukemia
Chronic Lymphocytic Leukemia = Chronic Lymphocytic Leukemia
Other Lymphocytic Leukemia = Other Lymphocytic Leukemia
Myeloid and Monocytic Leukemia = Acute Myeloid Leukemia, Acute Monocytic Leukemia, Chronic Myeloid Leukemia, Other Myeloid/Monocytic Leukemia
Acute Myeloid Leukemia = Acute Myeloid Leukemia
Acute Monocytic Leukemia = Acute Monocytic Leukemia
Chronic Myeloid Leukemia = Chronic Myeloid Leukemia
Other Myeloid/Monocytic Leukemia = Other Myeloid/Monocytic Leukemia
Other Leukemia = Other Acute Leukemia, Aleukemic, Subleukemic and NOS
Other Acute Leukemia = Other Acute Leukemia
Aleukemic, Subleukemic and NOS = Aleukemic, Subleukemic and NOS
Miscellaneous Malignant Cancer = Miscellaneous Malignant Cancer
In situ, benign or unknown behavior neoplasm = In situ, benign or unknown behavior neoplasm
Tuberculosis = Tuberculosis
Syphilis = Syphilis
Septicemia = Septicemia
Other Infectious and Parasitic Diseases including HIV = Human Immunodeficiency Virus (HIV) (1987+), Other Infectious and Parasitic Diseases
a - Do not use, not compatible with SEER incidence = Human Immunodeficiency Virus (HIV) (1987+)
b - Do not use, not compatible with SEER incidence = Other Infectious and Parasitic Diseases
Diabetes Mellitus = Diabetes Mellitus
Alzheimers (ICD-9 and 10 only) = Alzheimers (ICD-9 and 10 only)
Diseases of Heart = Diseases of Heart

Hypertension without Heart Disease = Hypertension without Heart Disease
Cerebrovascular Diseases = Cerebrovascular Diseases
Atherosclerosis = Atherosclerosis
Aortic Aneurysm and Dissection = Aortic Aneurysm and Dissection
Other Diseases of Arteries, Arterioles, Capillaries = Other Diseases of Arteries, Arterioles, Capillaries
Pneumonia and Influenza = Pneumonia and Influenza
Chronic Obstructive Pulmonary Disease and Allied Cond = Chronic Obstructive Pulmonary Disease and Allied Cond
Stomach and Duodenal Ulcers = Stomach and Duodenal Ulcers
Chronic Liver Disease and Cirrhosis = Chronic Liver Disease and Cirrhosis
Nephritis, Nephrotic Syndrome and Nephrosis = Nephritis, Nephrotic Syndrome and Nephrosis
Complications of Pregnancy, Childbirth, Puerperium = Complications of Pregnancy, Childbirth, Puerperium
Congenital Anomalies = Congenital Anomalies
Certain Conditions Originating in Perinatal Period = Certain Conditions Originating in Perinatal Period
Symptoms, Signs and Ill-Defined Conditions = Symptoms, Signs and Ill-Defined Conditions
Accidents and Adverse Effects = Accidents and Adverse Effects
Suicide and Self-Inflicted Injury = Suicide and Self-Inflicted Injury
Homicide and Legal Intervention = Homicide and Legal Intervention
Other Cause of Death = Other Cause of Death

Age recode MPS [Age recode with <1 year olds]
00-04 years = 00 years, 01-04 years
05-09 years = 05-09 years
10-14 years = 10-14 years
15-19 years = 15-19 years
20-24 years = 20-24 years
25-29 years = 25-29 years
30-34 years = 30-34 years
35-39 years = 35-39 years
40-44 years = 40-44 years
45-49 years = 45-49 years
50-54 years = 50-54 years
55-59 years = 55-59 years
60-64 years = 60-64 years
65-69 years = 65-69 years
70-74 years = 70-74 years
75-79 years = 75-79 years
80-84 years = 80-84 years
85+ years = 85+ years

Sex M/F [Sex]
Male = Male
Female = Female

Race (W/Unspec, B, O) [Race recode (White, Black, Other)]
Description: Caution should be exercised when using this variable. For more information, see http://seer.cancer.gov/seerstat/variables/seer/race_ethnicity.
White/Other unsp = White, Other unspecified (1978-1991)
Black = Black
Other (American Indian/AK Native, Asian/Pacific Islander) = Other (American Indian/AK Native, Asian/Pacific Islander)

Year of death (1973-2014) [Year of death]
1973-1974 = 1973, 1974
1975-1979 = 1975, 1976, 1977, 1978, 1979
1980-1984 = 1980, 1981, 1982, 1983, 1984

1985-1989 = 1985, 1986, 1987, 1988, 1989
1990-1994 = 1990, 1991, 1992, 1993, 1994
1995-1999 = 1995, 1996, 1997, 1998, 1999
2000-2004 = 2000, 2001, 2002, 2003, 2004
2005-2009 = 2005, 2006, 2007, 2008, 2009
2010-2014 = 2010, 2011, 2012, 2013, 2014

OUTPUT
Title: U.S. Mortality 1973-2014 (Nov 2016 sub), Race (WU/B/O), Event: COD rec (HIV grouped w/oth infectious)

These rates are provided for producing "All races combined" statistics.
Since populations are not available for other unspecified 1978-1991 race, these cases are grouped with whites in the rates.
There is no Unknown race in the mortality files, so to avoid excluding cases with Unknown race you must pick a race category for them on on the MP-SIR Rates tab.
Therefore, statistics for whites may be misleading.

Display Rates as: Cases per 100,000
Precision: 6 decimal places (default)

In the "case listing" session, select the following options:

Filename: suicide case listing Matrix-3
SEER*Stat Version: 8.3.4
Date: 19-Jan-18

Session Type: Case Listing

SUGGESTED CITATION
Software: Surveillance Research Program, National Cancer Institute SEER*Stat software (www.seer.cancer.gov/seerstat) version 8.3.4.
Data: Surveillance, Epidemiology, and End Results (SEER) Program (www.seer.cancer.gov) SEER*Stat Database: Incidence - SEER 18 Regs Research Data + Hurricane Katrina Impacted Louisiana Cases, Nov 2016 Sub (1973-2014 varying) - Linked To County Attributes - Total U.S., 1969-2015 Counties, National Cancer Institute, DCCPS, Surveillance Research Program, released April 2017, based on the November 2016 submission.

DATA
Database: Incidence - SEER 18 Regs Research Data + Hurricane Katrina Impacted Louisiana Cases, Nov 2016 Sub (1973-2014 varying) - Linked To County Attributes - Total U.S., 1969-2015 Counties

SELECTION
Select Only: Malignant Behavior, Known Age, Cases in Research Database

Case: {Cause of Death (COD) and Follow-up.COD to site recode} = 'Suicide and Self-Inflicted Injury' AND {Race, Sex, Year Dx, Registry, County.Year of diagnosis} = '1973-2014', '1973', '1974', '1975', '1976', '1977', '1978', '1979', '1980', '1981', '1982', '1983', '1984', '1985', '1986', '1987', '1988', '1989', '1990', '1991', '1992', '1993', '1994', '1995', '1996',

'1997', '1998', '1999', '2000', '2001', '2002', '2003', '2004', '2005', '2006', '2007', '2008', '2009', '2010', '2011', '2012', '2013', '2014'

TABLE
Column: Patient ID

COD to site recode
Age at diagnosis
Month of diagnosis
Year of diagnosis
Survival months
COD to site rec KM
Site recode ICD-O-3/WHO 2008
Site recode B ICD-O-3/WHO 2008
Primary Site
Primary Site - labeled
Race recode (White, Black, Other)
Sex
Age recode with <1 year olds
Derived AJCC Stage Group, 7th ed (2010+)
Derived AJCC T, 7th ed (2010+)
Derived AJCC N, 7th ed (2010+)
Derived AJCC M, 7th ed (2010+)
Derived AJCC Stage Group, 6th ed (2004+)
AJCC stage 3rd edition (1988-2003)
Total number of in situ/malignant tumors for patient
Sequence number
County
Race/ethnicity
Race recode (W, B, AI, API)
Insurance Recode (2007+)
Marital status at diagnosis
Health Service Area (NCI Modified)
First malignant primary indicator
SEER cause-specific death classification
Derived SS1977 (2004+)
Derived SS2000 (2004+)
Summary stage 2000 (1998+)
SEER historic stage A
SEER summary stage 2000 (2001-2003)
SEER summary stage 1977 (1995-2000)
RX Summ--Surg Prim Site (1998+)
RX Summ--Scope Reg LN Sur (2003+)
RX Summ--Surg Oth Reg/Dis (2003+)
Reason no cancer-directed surgery
Scope of reg lymph nd surg (1998-2002)
RX Summ--Reg LN Examined (1998-2002)
Surgery of oth reg/dis sites (1998-2002)
Site specific surgery (1973-1997 varying detail by year and site)

In the "incidence" session, select the following values:

Filename: Rate Session-1 Matrix-3
SEER*Stat Version: 8.3.4
Date: 28-Jan-18

Session Type: Rate

SUGGESTED CITATION
Software: Surveillance Research Program, National Cancer Institute SEER*Stat software (www.seer.cancer.gov/seerstat) version 8.3.4.
Data: Surveillance, Epidemiology, and End Results (SEER) Program (www.seer.cancer.gov) SEER*Stat Database: Incidence - SEER 9 Regs Research Data, Nov 2016 Sub (1973-2014) <Katrina/Rita Population Adjustment> - Linked To County Attributes - Total U.S., 1969-2015 Counties, National Cancer Institute, DCCPS, Surveillance Research Program, released April 2017, based on the November 2016 submission.

DATA
Database: Incidence - SEER 9 Regs Research Data, Nov 2016 Sub (1973-2014) <Katrina/Rita Population Adjustment> - Linked To County Attributes - Total U.S., 1969-2015 Counties

STATISTIC
Statistic: Age-Adjusted Rates (Age recode with <1 year olds) (2000 US Std Population (19 age groups - Census P25-1130) standard)

SELECTION
Select Only: Malignant Behavior, Known Age

Std Pop/Pop/Case: {Age at Diagnosis.Age recode with <1 year olds} = '00 years', '01-04 years', '05-09 years', '10-14 years', '15-19 years', '20-24 years', '25-29 years', '30-34 years', '35-39 years', '40-44 years', '45-49 years', '50-54 years', '55-59 years', '60-64 years', '65-69 years', '70-74 years', '75-79 years', '80-84 years', '85+ years', 'Unknown'

TABLE
Page: COD to site recode

Row: Site recode ICD-O-3/WHO 2008

Column: Age recode with <1 year olds

OUTPUT
Display Rates as: Cases per 100,000
Precision: 1 decimal place (default)

The output of these sessions is provided in Supplementary Data sets 1-3.

Supplementary Data Set 1. Suicide case listing 1973-2014

Supplementary Data Set 2. Suicide rate per age diagnosed

Supplementary Data Set 3. Suicide standardized mortality ratios after diagnosis
