# Supplementary material for: Suicide among cancer patients
Source: Nat Commun. 2019 Jan 14;10:207. doi: 10.1038/s41467-018-08170-1 (PMC6331593; doi:10.1038/s41467-018-08170-1)
Supplement: Supplementary file 6 — Description of Additional Supplementary Files [file 41467_2018_8170_MOESM6_ESM.docx]

**Title:** Supplementary Dataset 1
**Description:** Suicide case listing 1973-2014

**Title:** Supplementary Dataset 2
**Description:** Suicide rate per age diagnosed

**Title:** Supplementary Dataset 3
**Description:** Suicide standardized mortality ratios after diagnosis
